# Coexistence of pernicious anemia and prostate cancer - 'an experiment of nature' involving vitamin B_12 _modulation of prostate cancer growth and metabolism: a case report

**DOI:** 10.1186/1752-1947-3-9295

**Published:** 2009-11-24

**Authors:** Glenn Tisman, Seth Kutik, Christa Rainville

**Affiliations:** 1 Department of Nutrition and Neoplasia, Whittier Cancer Research Building, Bailey Street, Whittier, CA 90601, USA

## Abstract

**Introduction:**

This report presents the clinical and laboratory course of a patient with prostate cancer and severe vitamin B_12 _deficiency undergoing watchful waiting for prostate cancer. The possible interaction between therapy for B_12 _deficiency and the natural course of prostate cancer is presented.

**Case presentation:**

We present the case of a 75-year-old Chinese man with prostate cancer and pernicious anemia. His serum vitamin B_12 _level was 32 pg/ml (300-900 pg/ml) and holotranscobalamin was 0 pg/ml (>70 pg/ml). There was an unexpected rapid progression of Gleason's score during 10 months of watchful waiting. After the diagnosis of pernicious anemia was made, therapeutic injections of vitamin B_12 _were started. We observed a significant acceleration in prostate-specific antigen and prostatic acid phosphatase and a shortening of prostate-specific antigen doubling time after initiation of B_12 _therapy.

**Conclusion:**

We propose that the relatively short period of watchful waiting before histological progression of Gleason's score (GS [3+2] = 5 to GS [3+4] = 7 over 10 months) may have been a result of depleted holotranscobalamin 'active' B_12_. Replacement of B_12 _was associated with an initial rapid increase in serum prostate-specific antigen and prostatic acid phosphatase followed by stabilization. The patient represents an 'experiment of nature' involving vitamin B_12 _metabolism and raises the question as to whether rapid histological progression of Gleason's score was related to absence of serum holotranscobalamin while prostate-specific antigen and prostatic acid phosphatase, markers of cell growth, were accelerated by vitamin B_12 _replacement. To our knowledge, this is the first report of a possible cellular kinetic interaction between an epithelial malignancy and vitamin B_12 _metabolism.

## Introduction

Twenty percent of serum vitamin B_12 _is bound to a protein as holotranscobalamin (holoTC, 'active' B_12_) while 80% is bound to a glycoprotein as holohaptocorrin. B_12 _bound as holohaptocorrin is metabolically inert but represents the majority of measurable total serum B_12_. The most common cause of depressed holotranscobalamin is age-related gastric atrophy with hypochlorhydria leading to malabsorption of protein-bound B_12_. It is only rarely that patients present with classical pernicious anemia with the failure to secrete intrinsic factor. Our laboratory has previously reported on the frequency of depressed levels of holotranscobalamin in newly diagnosed patients with a variety of cancer types, including prostate cancer, and observed approximately 33% of all cancer patients (17/52) to be holotranscobalamin-insufficient before therapy [1]. In the same study, 5 out of 15 prostate cancer patients possessed deficient levels of holotranscobalamin [1]. B_12 _plays a critical role in DNA synthesis and one-carbon transfer reactions mediated through S-adenosylmethionine. B_12 _insufficiency may lead to decreased DNA methylation, which is associated with interference of histone metabolism and genetic mutation [2-4].

Pernicious anemia (PA) is associated with an increased rate of secondary malignancies, which include cancer of the stomach (standardized incident rate (SIR) = 2.9), esophagus (SIR = 3.2), and pancreas (SIR = 1.7) among men and women; myeloid leukemia among men (SIR = 1.8-5.2); and multiple myeloma among women (SIR = 2.5) [5]. An elevated incidence of secondary gastric carcinoid tumors was also reported. The risk of stomach cancer was highest in the first year after diagnosis of pernicious anemia (SIR = 7.4), but an increased risk persisted throughout life.

That vitamin B_12 _may accelerate normal and cancer cell proliferation within the milieu of B_12 _insufficiency may be observed in tissue culture for L1210 mouse leukemia cells grown in a B_12 _deficient medium [6] as well as in the clinic [7]. The administration of parenteral B_12 _in pancytopenic patients with B_12 _deficiency is associated with a brisk hematopoietic response. In such patients, associated epithelial cell dysplasias (bronchial, esophageal, buccal and cervical) return towards normal [8]. In one study of 51 patients with bronchial metaplasia, 77% of the control group remained stable while 90% of patients treated with folate and B_12 _decreased in metaplasia scoring, which demonstrated a reversal of metaplasia [9]. Additionally, Corsino *et al*. demonstrated kinetic control of chronic myelogenous leukemia cells in response to parenteral replacement of B_12 _in a patient with both pernicious anemia and chronic granulocytic leukemia [7]. They described this in their paper as 'an experiment of nature'.

Cognizant of the role B_12 _played in dysplasia and normal and cancer cell kinetics, we closely monitored potential modulator effects of B_12 _replacement therapy in a patient presenting with prostate cancer and advanced B_12 _depletion whose clinical course of watchful waiting had been remarkably short.

## Case presentation

A 75-year-old Chinese man presented to his urologist with prostate-specific antigen (PSA) = 4 and this was followed by core needle biopsy. The diagnosis of clinical T1c, Gleason's score (GS) = [3+2] = 5 adenocarcinoma of the prostate was made. One of six cores (5% of the length) contained adenocarcinoma and two of six cores contained grade III prostatic intraepithelial neoplasia (PIN). The patient elected to undergo no further therapy other than observation. Unfortunately, a baseline complete blood count (CBC) was not obtained at the time of prostate biopsy on 18 October 2007. A hemoglobin (Hb) level of 13.7 g/dl and a mean corpuscular volume (MCV) of 99 fl were recorded on the 19 August 2005. Over the next 10 months, he had no urological complaints; however, repeat PSA measurements revealed rapidly progressive disease with an increase in his PSA from 4.0 to 16.7 ng/ml and a decrease in prostate-specific antigen doubling time (PSADT) from 10.5 to 5.3 months.

The patient's urologist repeated the prostate biopsy on 23 August 2008 and noted clinical progression from T1c to T2b disease. The biopsy revealed 4 of 6 cores positive for GS = [3+4] = 7 with 25% of the submitted material containing cancer.

The patient presented to our office and was noted to be anemic, but with no specific complaints of tiredness. Anemia work-up revealed Hb at 7.5 g/dl and MCV at 124 fl. There was macro-ovalocytosis, hypersegmentation of neutrophils, and a single six lobe neutrophil on a Wright's Giemsa stained blood smear. The serum level of total B_12 _was 32 pg/ml (= 300 pg/ml), holoTC was 0 pg/ml (>70 pg/ml), serum methylmalonic acid (MMA) was 13,970 nM/l (87-318 nM/l), total homocysteine (tHcy) was 108.8 μM/l (<12 μM/l), reticulocyte count was depressed to 9500 cells/μl (25,000-90,000 cells/μl) and haptoglobin was <6 mg/dl (43-212 mg/dl). Blocking antibody to intrinsic factor was present. A diagnosis of B_12 _deficiency due to presumed atrophic gastritis and classical pernicious anemia was made. Iron deficiency was ruled out by virtue of normal levels for serum iron/total iron binding capacity (TIBC), % saturation of TIBC, ferritin, and red cell zinc protoporphyrin. Serum folic acid was 17.4 ng/ml (=5). Other causes of anemia were excluded through a negative Coomb's test and normal renal function as manifested by normal serum cystatin-c and serum creatinine. Thyroid antibody studies plus serum thyroid stimulating hormone (TSH) and free T4 were normal. There were no neurological findings, proprioception of the toes and vibratory sensation of the toes to a 256 vps tuning fork were intact. Diagnoses of laboratory, clinical and histological progression of prostate cancer in addition to profound pernicious anemia were confirmed.

**Figure 1:**
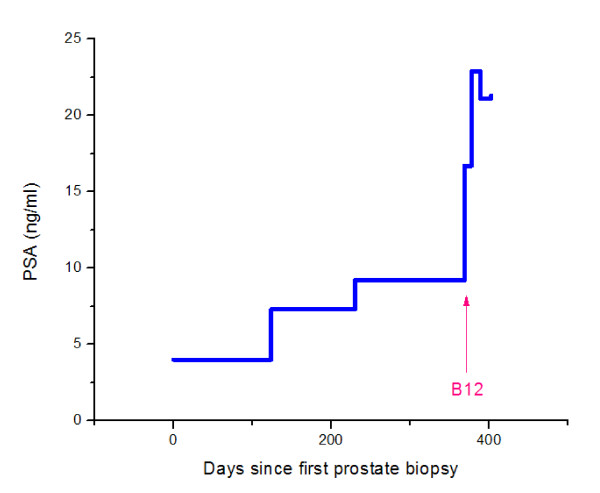
**Prostate specific antigen measurements**.

**Figure 2:**
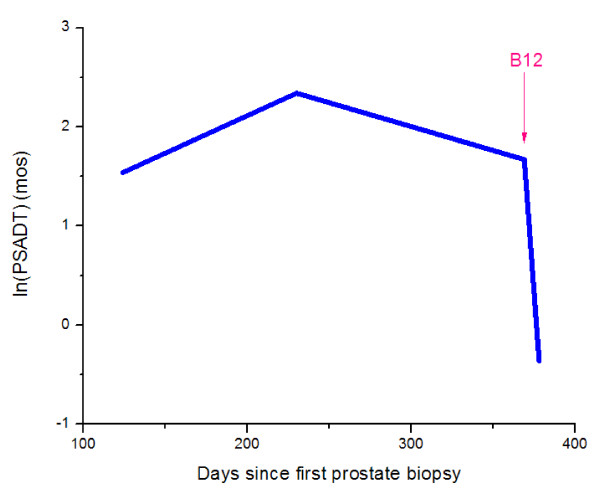
**Prostate specific antigen doubling time in months measurements**.

All PSA and prostatic acid phosphatase (PAP) measurements were made with the same in-house DPC Immulite^® ^assay with sensitivity to 0.003 ng/ml, standard error of 0.003 ng/ml and coefficient of variation (CV) <4%. The patient was started on subcutaneous B_12 _injections as cyanocobalamin 1000 μg daily while measurements for methylmalonic acid, total homocysteine, reticulocyte count, Hb, PSA, PSADT, PSA velocity (PSAV), and PAP, were recorded at frequent intervals (Table 1 and Figures 1 and 2). The patient did not receive supplemental folic acid; however, he was given an oral preparation of potassium chloride 20mEq daily anticipating a possible intracellular potassium shift in response to B_12 _administration.

**Table 1:** Clinical laboratory data

	18 October 2007 - Diagnosis prostate cancer	19 February 2008 - PSA progression	4 June 2008 - PSA progression	21 October 2008 - Diagnosis of PA	30 October 2008 - B_12 _Rx	10 November 2008 - B_12 _Rx	24 November 2008 - B_12 _Rx
Hb, g/dl	-	-	-	7.5	8.6	9.2	9.9
MCV, fl	-	-	-	124	122	117.7	113
Reticulocyte count, cells/μl	-	-	-	9500	23,230	11,450	7470
MMA, nM/l	-	-	-	13,970	3069	-	290
tHcy, μM/l	-	-	-	109	14	18.8	14.8
B_12_, pg/ml	-	-	-	32	>1500	>1500	>1500
holoTC, pg/ml	-	-	-	0	-	-	-
Testosterone, ng/dl	-	-	-	331	346	205	323
PSA, ng/ml	4.0	7.3	9.2	16.7	22.9	21.1	21.3
PAP, ng/ml	-	-	-	1.5	2.3	3.2	2.4
Folate, ng/ml	-	-	-	17.4	-	17.5	-
B_12 _injection, μg/day subcutaneous	-	-	-	1000	1000	1000	1000

B_12_, cobalamin; Hb, haemoglobin; holoTC, holotranscobalamin; MMA, methylmalonic acid; PA, pernicious anemia; PAP, prostatic acid phosphatase; PSA, prostate specific antigen; Rx, treatment; tHcy, total homocysteine.

**Figure 3:**
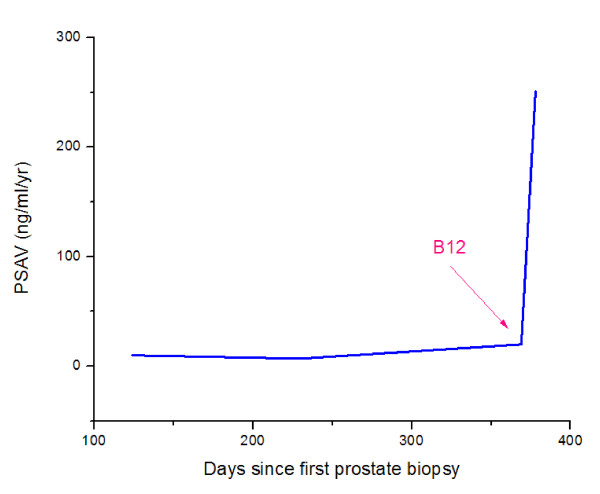
**Prostate specific antigen velocity measurements**.

**Figure 4:**
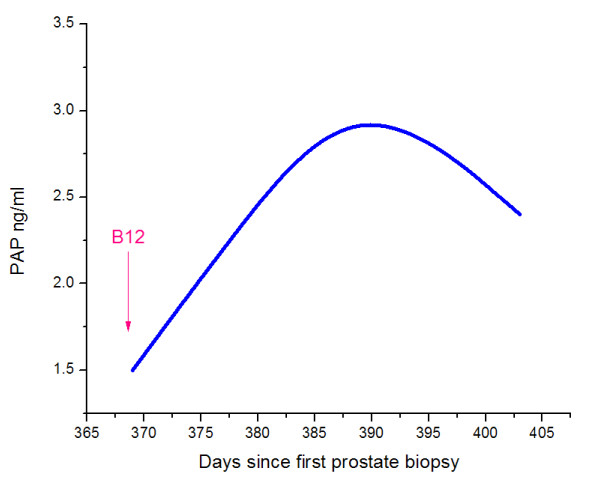
**Prostatic acid phosphatase measurements**.

The patient had a hematological response to B_12 _replacement. Following B_12 _supplementation, we recorded a rapid increase in serum PSA from 16.7 to 22.9 ng/ml over just a 9-day period and PAP increased from 1.5 ng/ml to 2.3 ng/ml and ultimately to 3.2 ng/ml (<3.0 ng/ml) 20 days after the initiation of B_12 _(Table 1 and Figures 1, 2, 3, and 4). Twenty days after initiation of B_12_, PSA and PAP levels stabilized at 21.1 ng/ml and 3.2 ng/ml, respectively (Table 1). After 34 days of daily B_12 _injections, PSA was 21.3 ng/ml and PAP was 2.4 ng/ml (Table 1). PSAV was noted to have rapidly increased from 19.7 ng/ml/year to 251 ng/ml/year while PSADT decreased from 5 months to 0.65 months.

## Discussion

We conjecture that this patient's accelerated prostatic histological and clinical progression during watchful waiting could have been modulated by the concomitant profound deficiency of B_12 _in light of the central role played by B_12 _in DNA synthesis, association with worsening of epithelial dysplasia and induction of genetic mutations. Data from studies by Choo and colleagues reveal that the usual rate of histological progression for watchful waiting is 2.4% of patients at a median of 29 months of observation [10,11]. Histological progression is generally limited to one higher Gleason's grade [10,11]. Our case progressed two Gleason's grades within 10 months while clinical stage advanced from T1c to T2b.

The same expert pathologist ascertained the Gleason score progression through a side-by-side comparison of the first biopsy with the second. Furthermore, it should be noted that 78% of the time, intrapathological review is precisely reproducible and 87% of the time reproducible within +1 Gleason score [12]. However, there is always the possibility that a needle sampling variation of cancer tissue could have accounted for the apparent histological progression. However, the rapid change of clinical stage (T1c to T2b), the increase in number to positive cores (1 of 6 to 4 of 6) and the accelerated PSA and PAP kinetics suggest there could be a B_12 _prostate cancer interaction.

At the same time, a rapid rise in both PSA and PAP, acceleration of PSAV and shortening of PSADT rapidly followed supplementation of B_12_. These changes suggest that absence of holoTC, the active component of serum B_12_, may have in part retarded prostate cancer growth while at the same time possibly participating in generation of mutations associated with rapid histological progression.

## Conclusion

We recommend that all patients opting to pursue watchful waiting for prostate cancer undergo an initial serum B_12 _assay, including serum holoTC. HoloTC is depleted before total B_12 _falls within the abnormal range [1]. Although our findings are preliminary and based on only a single case report, monitoring a patient's B_12 _and holoTC remain important due to the prevalence of B_12 _and holoTC insufficiency in elderly individuals for whom watchful waiting is frequently implemented [1]. If B_12 _insufficiency is present then it should be corrected immediately. The AxSYM^® ^Active-B_12 _holotranscobalamin via the Abbott AxSYM immunoassay is the only Food and Drug Administration approved assay for holoTC in the USA. Glass adsorption assay is another technique (developed in this laboratory [13] as a modification of that first described by Victor Herbert).

## Abbreviations

B_12_: cobalamin; CBC: complete blood cell count; GS: Gleason score; Hb: haemoglobin; holoTC: holotranscobalamin; MCV: red blood cell mean corpuscular volume; MMA: methylmalonic acid; PA: pernicious anemia; PAP: prostatic acid phosphatase; PIN: prostatic intraepithelial neoplasia; PSA: prostate specific antigen; PSADT: PSA doubling time in months; PSAV: PSA velocity; SIR: standardized incident ratio; tHcy: total homocysteine; TIBC: total iron binding capacity

## Consent

Written informed consent was obtained from the patient for publication of this case report and any accompanying images. A copy of the written consent is available for review by the Editor-in-Chief of this journal.

## Competing interests

The authors declare that they have no competing interests.

## Authors' contributions

GT analyzed and interpreted the patient data regarding the pernicious anemia and prostate cancer. SK addressed the reviewers' comments and contributed analysis and review of B_12 _metabolism as relates to his current research in holotranscobalamin in cancer patients. CR completed a thorough review of other pertinent papers in the literature and reviewed mathematical analysis and gathered factual patient data as pertained to this case report. All authors read and approved the final manuscript.
